# Supplementary material for: Regulatory Effects of ABA and GA on the Expression of Conglutin Genes and LAFL Network Genes in Yellow Lupine (Lupinus luteus L.) Seeds
Source: Int J Mol Sci. 2023 Aug 3;24(15):12380. doi: 10.3390/ijms241512380 (PMC10418516; doi:10.3390/ijms241512380)
Supplement: Supplementary file 1 [file ijms-24-12380-s001.zip › ijms-2507963-supplementary.pdf]

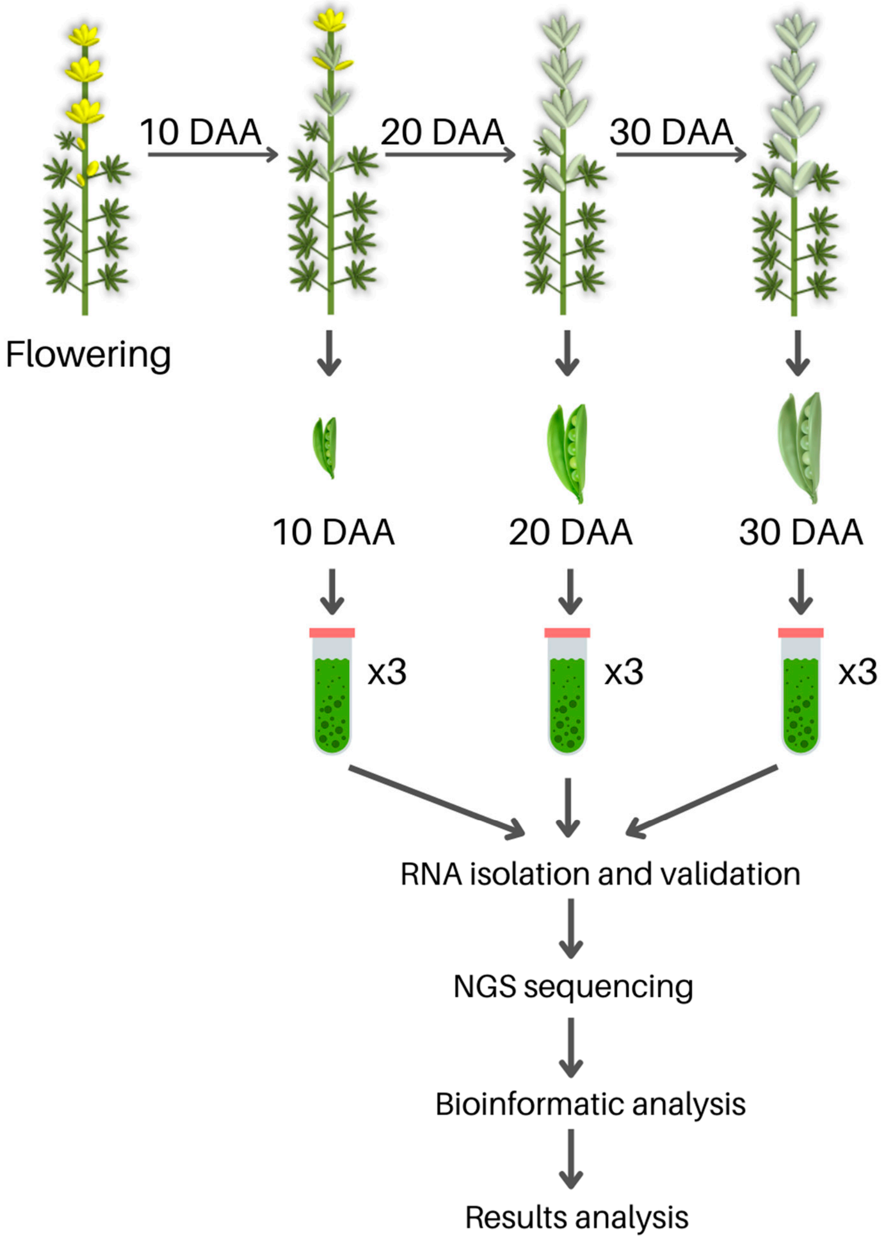

Figure S1: Schematic of the methodology and experimental setup used in the RNA-seq experiment

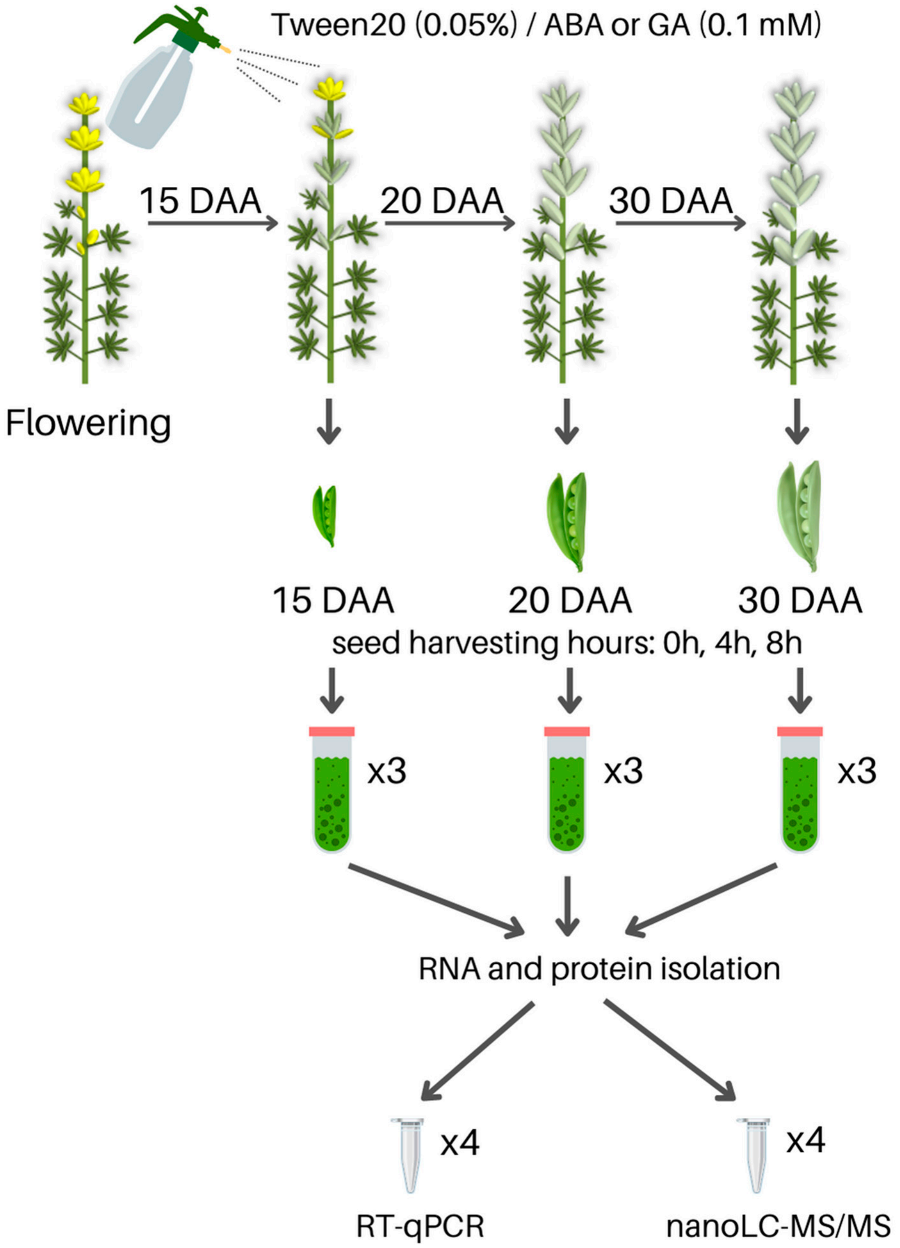

Figure S2: Schematic of the methodology and experimental set-up used to examine the effects of ABA and GA on the expression of selected genes and the amount of accumulated stor-age proteins
